# Screening and identification of the tumor‐associated antigen CK10, a novel potential liver cancer marker

**DOI:** 10.1002/2211-5463.12122

**Published:** 2017-03-21

**Authors:** Zhaoyu Zhu, Zheng Liu, Yuchun Liu, Chunyan Wang, Ruyu Li, Hui Liu, Baohua Gu, Guodong Li, Shoutao Zhang

**Affiliations:** ^1^ School of Life SciencesZhengzhou UniversityChina; ^2^ Xinyang Vocational and Technical CollegeXinyangChina; ^3^ Henan Key Laboratory of Bioactive MoleculesZhengzhou UniversityChina; ^4^ Collaborative Innovation Center of New Drug Research and Safety EvaluationZhengzhouChina

**Keywords:** antibody, biomarker, hepatocellular carcinoma, tumor‐associated antigen

## Abstract

Hepatocellular carcinoma (HCC) is a malignancy that is associated with high mortality rates in Asia. These tumors are highly invasive and their etiology is frequently unknown. Thus, most patients are diagnosed in the middle and late stages of the disease, and thus do not have sufficient time for therapy. Therefore, it is essential to study the early diagnosis and treatment of HCC; in this regard, the study of tumor‐associated antigens has received much attention. Here, antigens from the human primary HCC cell line, QGY‐7703, were used to immunize mice in order to prepare monoclonal antibodies. The specific antigen recognized by antibody 11C3 was purified from total protein lysates of QGY‐7703 by immunoaffinity chromatography. The validity of the candidate antigen as a new HCC‐associated marker was tested using SDS/PAGE, western blot, HPLC‐ESI‐MS/MS, and RT‐qPCR. Our results showed that the levels of CK10 in HCC‐derived cell lines were significantly higher than those in normal liver cells. Thus, we suggest that CK10 may be involved in the formation and development of HCC, and may be a therapeutically targetable tumor‐associated antigen.

**Abbreviations**
AFP: alpha fetoprotein
BCA: bicinchoninic acid
CK10: cytokeratin 10
DMEM: dulbecco's modified eagle medium
HCC: hepatocellular carcinoma
HRP: horseradish peroxidase
mAb: monoclonal antibody
RIPA: radio immunoprecipitation assay
TAA: tumor‐associated antigen
TMB: 3,3′,5,5′‐Tetramethylbenzidine

Hepatocellular carcinoma (HCC) is one of the most common malignancies worldwide, with an unusually high occurrence in China, especially in South China 1, 2. Hepatitis B virus (HBV) or hepatitis C virus (HCV) infection, dietary exposure to aflatoxin, and alcohol drinking are the principal etiological factors for HCC 3, 4. HCC is a highly malignant, invasive, and fast‐growing disease, which is also associated with high rates of recurrence and fatality. Indeed, the majority of people with HCC die within 1 year of disease detection. The high fatality rate can in part be attributed to a lack of diagnostic methods that allow early detection 5. HCC is often diagnosed at an advanced stage when potentially curative therapies, including resection, transplantation, and percutaneous/transarterial interventions, are of limited efficacy. In order to reduce the morbidity and mortality associated with HCC, the establishment of early diagnosis and development of novel systemic therapies for advanced disease stages, including drugs, gene and immune therapies as well as primary HCC prevention, are of paramount importance 6, 7.

Identification of HCC‐associated antigens by molecular and immunological approaches has provided opportunities for the development of sensitive and specific diagnoses and immunotherapies 6. The immune system can recognize antigenic changes in cancer cells, and further develop autoantibodies against these so‐called ‘tumor‐associated antigens’ (TAAs) 7, 8. These antigens are often products of mutated cellular genes, aberrantly expressed normal genes, or genes encoding viral proteins 9. A wide variety of TAAs have been identified using corresponding mAbs, and a number of cancer therapies using mAbs specific for TAAs have been attempted 10, 11, 12.

An HCC‐associated antigen, alpha fetoprotein (AFP), is a primary tumor marker for HCC and has been used to detect and monitor HCC 13, 14. However, in early‐stage HCC patients, the false‐negative rate attributable to monitoring AFP level alone may be as high as 40%; thus, its sensitivity and specificity are not optimal 15. Des‐γ‐carboxyprothrombin is also an HCC‐associated antigen that is used to detect HCC, and its serum levels increase in 55–74% of patients with HCC; however, the measurement of des‐γ‐carboxyprothrombin level alone is not sufficient for early detection of HCC 16, 17. Therefore, the identification of more specific and sensitive HCC‐associated markers will contribute to improvements in HCC early diagnosis and treatment.

Monoclonal antibodies are useful tools for the analysis of antigens that might have clinical applications in the diagnosis and immunotherapy of HCC. In this study, antigens derived from the human primary HCC cell line, QGY‐7703, were used to immunize mice for the production of monoclonal antibodies. A specific antigen identified by antibody 11C3 was purified from total protein lysates of QGY‐7703 by immunoaffinity chromatography (IAC). The antigen was then further characterized by SDS/PAGE, western blot, HPLC‐ESI‐MS/MS, and RT‐qPCR in order to determine whether it could be a novel HCC‐associated antigen. We found that the expression of both CK8 and CK10 was significantly higher in HCC cells than in their normal counterparts. Since CK10 in particular was highly upregulated, we suggest that it may be involved in the formation and development of HCC.

## Materials and methods

### Cell lines and cell culture

BALB/c mice were supplied by Zhengzhou Autobio Co., Ltd (Zhengzhou, China). The human primary hepatocellular carcinoma cell line QGY‐7703 was a kind gift from Dr. Yingle Liu of Wuhan University. Human normal liver cell line L02 was supplied by Zhengzhou Autobio Co., Ltd. All cell lines were cultured in Dulbecco's modified eagle medium (DMEM) (Solarbio, Beijing, China) supplemented with 10% fetal bovine serum (Sijiqing Biological Engineering, Hangzhou, China), 100 U·mL^−1^ penicillin, and 100 μg·mL^−1^ streptomycin in an incubator with a humidified atmosphere (5% CO_2_) at 37 °C.

Written informed consent was obtained for all subjects according to the Declaration of Helsinki and the General Assembly Revision of 2008. The study has been approved by the Ethics Committee of Zhengzhou University.

### mAb preparation

The mAb against human HCC cells was prepared using the hybridoma technique; the hybridoma cell line was obtained by fusion of SP2/0 myeloma cells with spleen cells from BALB/c mice (8–10‐week‐old females) immunized with QGY‐7703 cells. The hybridoma cell strain was injected into the abdominal cavity of BALB/c mice, and the ascites were collected 2 weeks later; purification through a protein A affinity column was then performed. CK10 and CK8 monoclonal antibodies were purchased from Abcam (Cambridge, UK).

### ELISA analysis

Indirect ELISA was used to determine the specificity of the 11C3 antibody. The cell culture plate was packaged using the following procedure: single cell suspensions were obtained from HCC cells (QGY‐7703) and from human normal liver cells (L02) by trypsin digestion, and adjusted to a density between 1 × 10^5^ and 1 × 10^6^ cells·mL^−1^ by dilution with 20% DMEM. The cells were then added (50 μL per well) to 96‐well plates and incubated for 24 h (37 °C, 5% CO_2_). Culture medium was removed and cells were washed three times with PBS (pH 7.4). Blocking solution (100 μL of 1% BSA per well), was added and cells were incubated at 4 °C for 12 h. This was followed by washing three times with PBS.

Indirect ELISA analysis was carried out using the following procedure. The 11C3 antibody was diluted to 5 × 10^−3^ mg·mL^−1^ with antibody diluent, and 50 μL was added to each well. Plates were covered to minimize evaporation, and antibody incubation was carried out for 30 min at 37 °C. After antibody removal, wells were washed three times with wash buffer (PBS containing 0.05% Tween‐20). HRP‐goat anti‐(mouse IgG) (50 μL) was added to each well for 30 min at 37 °C, then samples were washed three times with wash buffer. TMB substrate solution (100 μL per well) was incubated at 37 °C for 10 min before addition of 100 μL per well of stop solution. Plates were then read at 490 nm using an enzyme‐linked analyzer.

### Preparation of total cellular protein

The culture medium was removed by aspiration and cells were washed three times with ice‐cold PBS. One milliliter of ice‐cold RIPA lysis buffer (supplemented with protease inhibitors) was added per 25 cm^2^ of labware area, and culture bottles were agitated to ensure that all of the cells were covered with the lysis buffer. Following incubation on ice for 30 min, lysates were transferred to microcentrifuge tubes, and DNA was fragmented by repeated passage through a syringe fitted with a 12G needle. After centrifugation at 12 000 rpm for 5 min at 4 °C, the supernatant was used for subsequent experiments. The total cellular protein concentration was determined with a bicinchoninic acid kit (Life Technologies, Carlsbad, CA, USA).

### Western blotting analysis

Total cellular protein lysates were resolved in SDS/PAGE gels, transferred to nitrocellulose membrane, and blocked with blocking buffer (PBS containing 5% nonfat dry milk and 0.05% Tween‐20, pH 7.4), incubated with diluted 11C3 antibody for 1 h. This was followed by three 5‐min washes in TBS‐T and incubation with goat anti‐(mouse IgG)‐HRP (Santa Cruz Biotechnology, Santa Cruz, CA, USA). The bands were then visualized with the ECL plus system.

### Preparation of antibody affinity chromatography column

The 11C3 antibody was dissolved in coupling buffer (0.1 m NaHCO_3_ pH 8.3 containing 0.5 m NaCl). The required amount of CNBr‐activated Sepharose 4B was weighed out, then swollen and washed with 1 mm HCl. The coupling solution containing the 11C3 antibody was mixed with medium in a stoppered vessel and rotated end‐over‐end overnight at 4 °C; other gentle stirring methods may be employed. Excess ligand was removed by washing with at least five medium (gel) volumes of coupling buffer, and the percolate was collected in order to calculate the antibody coupling rate. Any remaining active groups were blocked. The medium was then transferred to a 0.1 m Tris‐HCl buffer, pH 8.0, and solutions were left to stand for 2 h. The medium was then washed with at least three cycles of buffers with alternating pH (see below), and at least five medium volumes of each buffer were used.

Each cycle consisted of a wash with 0.1 m acetate buffer, pH 4.0, containing 0.5 m NaCl followed by a wash with 0.1 m Tris‐HCl, pH 8, containing 0.5 m NaCl. The medium coupled with antibody should be stored in 20% anhydrous ethanol at −20 °C.

### IAC purification of antigen

The coupling solution containing the QGY‐7703 total protein was mixed with antibody coupling medium in a stoppered vessel, and rotated end‐over‐end for 24 h at 4 °C. Media was pipetted into a 2‐mL purification column. Purification of antigen was performed using the AKTA prime plus (GE Healthcare, Stockholm, Sweden) employing the purification column. Impure protein was first eluted by PBS (pH 7.4), and the antigen was then eluted in elution buffer (0.1 m Gly‐HCl, pH 2.0), and immediately neutralized by Tris‐HCl pH 9.0. Eluted antigen was collected, neutralized with Tris‐HCl pH 9.0.

The protein solution was concentrated by TCA precipitation and then analyzed by SDS/PAGE and western blot.

### Mass spectrometry and western blotting identification of antigen

After determining the molecular weight of the purified antigen protein by SDS/PAGE, bands were excised from the gel and were digested with trypsin, then identified by liquid chromatography‐electrospray spectrometry‐mass/mass spectrometry (LC‐ES‐MS/MS). The tandem mass spectrum data from LC‐ES‐MS/MS were used to identify the proteins through matching with theoretical mass spectrum data from the IPI_humanv.3.87 database.

We used commercial CK10 and CK8 monoclonal antibodies (Abcam) to further characterize the purified antigens using the western blotting method described above.

### RT‐PCR analysis

Total RNA was extracted from cancer cell lines and normal cell lines. The cDNA was generated by a reverse transcription‐PCR method using the PrimeScript RT‐PCR kit. Quality of the cDNA was confirmed by PCR of GAPDH. Gene‐specific PCR primers used to amplify CK1, CK10, CK9, CK8, and GAPDH (CK1 sense: 5′‐AGGATGTGGATGGTGCTTAT‐3′, antisense: 5′‐GCTTTGCTCTTCTGGGCTAT‐3′, size: 235 bp; CK10 sense: 5′‐TGCCAACATCCTGCTTC‐3′, antisense: 5′‐AGCCTTGGTCAGGGTCA‐3′, size: 151 bp; CK9 sense: 5′‐GAGGTGATGGTGGTATT‐3′, antisense: 5′‐TTATCCAAGTAAGAGGC‐3′, size: 83 bp; CK8 sense: 5′‐GGCATCACCGCAGTTAC‐3′, antisense: 5′‐AGGCTCCACTTGGTCTC‐3′, size: 191 bp; GAPDH sense: 5′‐GCACCGTCAAGGCTGAGAAC‐3′, antisense: 5′‐TGGTGAAGACGCCAGTGGA‐3′, size: 137 bp). The primers were designed by ourselves and purchased from GENEWIZ. The RT‐PCR assay kits were purchased from TaKaRa. The PCR reaction was carried out under the following conditions: 95 °C for 3 min, 95 °C for 10 s, 50 °C for 30 s, 72 °C for 30 s, for a total of 35 cycles, followed by 72 °C for 10 s, and 15 °C hold. PCR products were then analyzed by running a 2% gel electrophoresis.

### Real‐time quantitative PCR analysis

The RT‐qPCR reaction was carried out under the following conditions: 95 °C for 3 min, 95 °C for 10 s, 50 °C for 30 s, 72 °C for 30 s, for a total of 40 cycles, followed by a 15 °C hold. RT‐qPCR products were then analyzed by running a 2% gel electrophoresis.

## Results

### Hybridoma cell preparation and the clonal selection

The serum titer produced following immunization of mice was determined by ELISA. The endpoint titer was defined as the highest dilution at which the OD_450_ was 2.1‐fold higher than that of wells receiving control serum. As shown in Table 1, the titer of immunized mice serum reached 6.4 × 10^4^ or more, which meets the requirements of cell fusion.

**Table 1:** The titer of immunized mice serum

Mice line	1/200	1/1K	1/2K	1/4K	1/8K	1/16K	1/32K	1/64K	1/128K	1/256K
1#	2.949	2.842	2.488	2.038	1.738	1.229	0.812	0.474	**0.283**	0.177
2#	3.107	2.605	2.676	2.123	1.639	1.148	0.713	0.39	**0.241**	0.147
3#	2.955	2.811	2.633	1.848	1.424	0.916	0.634	**0.347**	0.188	0.121
4#	3.099	2.599	2.51	2.512	1.967	1.459	0.968	0.559	**0.316**	0.175
5#	3.125	2.669	2.113	1.89	1.495	1.001	0.648	0.372	**0.204**	0.136
Negative control	0.496	0.408	0.052	0.057	0.055	0.05	0.059	0.061	0.1	0.082

Serum dilution times is antibody titer. Antibodies were defined as positive when they yielded an absorbance with an OD_450_ that was 2.1‐fold higher than that found in control serum.

After cell fusion and ELISA detection, we identified five cell lines that produced an OD_450_ of more than 0.5. These lines were cloned in culture to give five strains of stable hybridoma cell lines secreting monoclonal antibody, which were named 11D6B4C2, 11H8F5G9, 11D11E10C3, 14B11A3G2, and 12E12F9G6.

### Monoclonal antibody subtype detection results

The OD values under the 490 nm were then measured. As shown in Table 2, this revealed that the Ig subtypes of cell lines 11D6B4C2, 11H8F5G9, 11D11E10C3, and 14B11A3G2 were IgG1, and that of 12E12F9G6 was IgM.

**Table 2:** The subtype testing of monoclonal antibody

Cell line	IgG1	IgG2a	IgG2b	IgG3	IgA	IgM
11D6B4C2	0.423	0.054	0.047	0.046	0.043	0.041
11H8F5G9	0.32	0.04	0.044	0.041	0.043	0.042
11D11E10C3	0.737	0.046	0.043	0.041	0.04	0.044
14B11A3G2	0.616	0.04	0.044	0.043	0.041	0.046
12E12F9G6	0.038	0.043	0.04	0.035	0.036	0.254

### Purification of monoclonal antibodies

**Figure 1:**
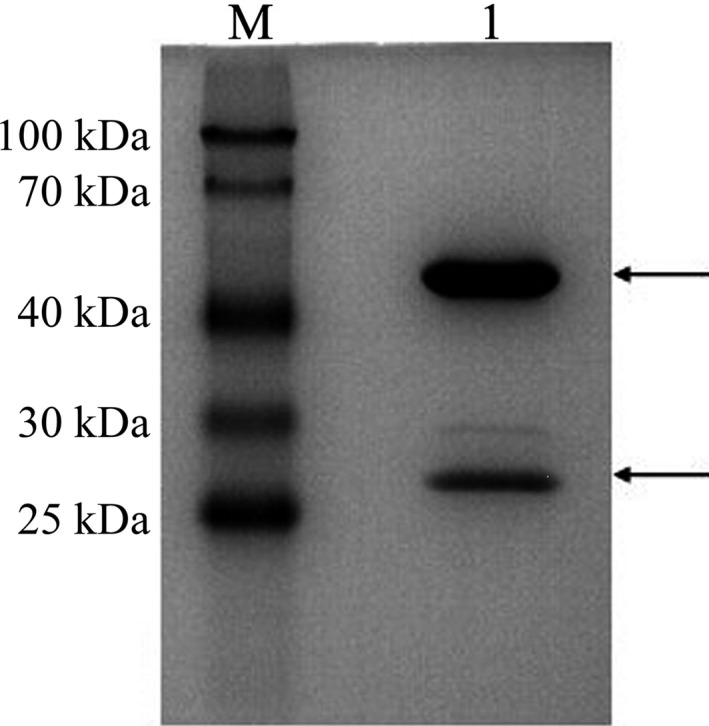
SDS/PAGE of monoclonal antibody 11C3 purified by Protein A.

We selected a strongly positive hybridoma cell line (clone No. 11D11E10C3) that stably secreted mAb and named it 11C3 for the follow‐up study. SDS/PAGE showed that the purified mAb 11C3 possesses only two strips: the heavy and the light chains of IgG. The molecular weight of the heavy chain is approximately 55 kDa, while the light chain is about 26 kDa, consistent with the theoretical value of murine IgG heavy and light chains. The concentration of monoclonal antibody was 5 mg·mL^−1^ (Fig. 1).

### Binding capacity of mAb 11C3 with hepatoma cells and normal liver cells

An indirect two‐step method of ELISA was applied to compare the binding capacity of mAb 11C3 to hepatoma cells and normal liver cells. This revealed that, although mAb 11C3 can bind to both cell types, the capacity of binding to hepatoma cells was significantly higher than that observed with normal liver cells (Table 3).

**Table 3:** ELISA analysis of 11C3

Cell line	OD value		
	Replicas hole 1	Replicas hole 2	Replicas hole 3
QGY‐7703	1.158	1.437	1.282
L02	0.186	0.081	0.137

**Figure 2:**
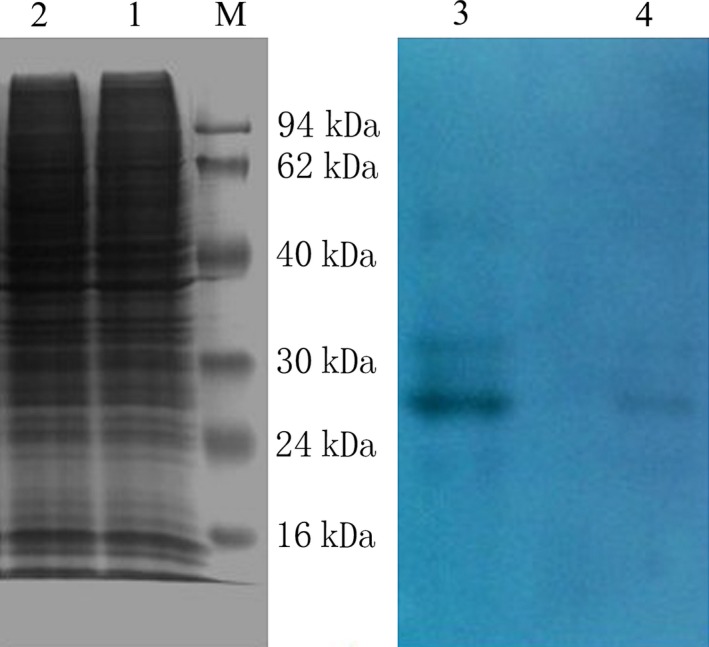
SDS/PAGE and western blotting analysis of total cellular protein from QGY‐7703 human hepatocellular cancer cells and L02 human normal liver cells. Lane M, marker; Lane 1, total cellular protein of QGY‐7703 cells; Lane 2, total cellular protein of L02 cells; Lane 3, western blotting of QGY‐7703 total cellular protein; Lane 4, western blotting of L02 total cellular protein.

While both in well growing condition and after reaching confluence, the numbers of cell strains were similar, and high efficient RIPA lysates could be introduced to extract total cellular protein. Bicinchoninic acid tests showed that the concentration of total protein extract was closer, both around 2.3 mg·mL^−1^. Results of western blot showed that the amount of 11C3 antigen expression in hepatoma cells is higher than in normal liver cells L02. The results are consistent with the ELISA results (Fig. 2).

### Purification of the candidate antigen

**Figure 3:**
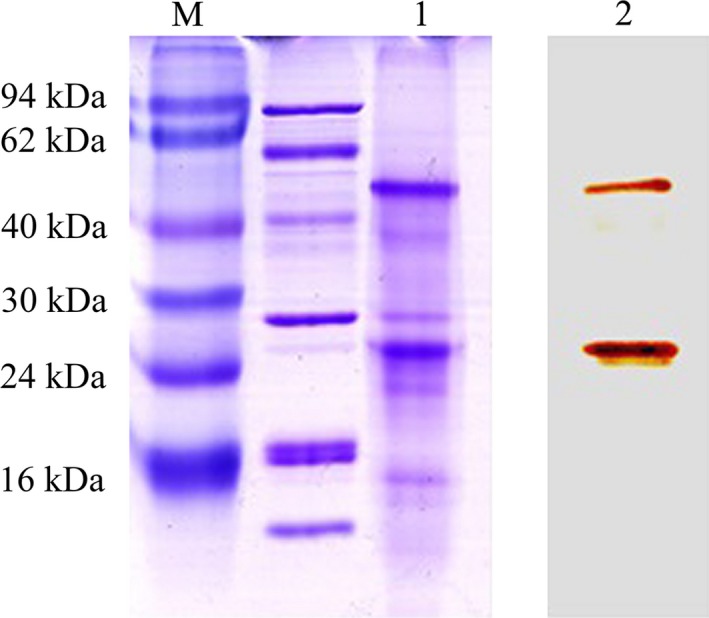
SDS/PAGE and western blotting analysis of the purified antigen of 11C3. Lane M, marker; Lane 1, concentration of elution peak by immunoaffinity chromatography; Lane 2, western blotting of antigen 11C3 purified by immunoaffinity chromatography.

The SDS/PAGE results showed that two prominent bands with molecular weights of ~55 kDa and ~28 kDa were obtained from the total protein lysates of QGY‐7703. Western blotting showed that these two bands react specifically with mAb 11C3, confirming that they are the antigens recognized by mAb 11C3 (Fig. 3).

### Mass spectrometry identification of protein bands

The two protein bands above were excised from the SDS/PAGE gel. Following tryptic digestion and processing, bands were analyzed using 5600 Triple TOF LC‐ESI‐MS/MS. Results were compared to the IPI_humanv.3.87 database using Mascot. We list here the top five scored matches along with the information on these proteins. Tables 4 and 5 show the results for the 55 kDa and 28 kDa gel strips, respectively. Proteins with the top scoring matches of the two gel strips are very similar and are nearly all keratin. Previous studies have showed that some cytokeratins are related to tumorigenesis, and are specifically expressed in tumor tissues 18, 19, 20, 21, 22, 23, 24, 25, 26.

**Table 4:** Proteins match with protein gel strips 1 (about 55 KD)

Protein ID	Protein Score	Protein mass	Coverage	Unique peptide	Unique spectra	Description
IPI00220327	2581.04	66170.07	0.441	25	51	CK1
IPI00009865	2328.47	59019.78	0.449	24	53	CK10
IPI00019359	2159.15	62254.9	0.568	24	54	CK9
IPI00554648	1839.61	53671.13	0.159	8	16	CK8

**Table 5:** Proteins match with protein gel strips 2 (about 28 KD)

Protein ID	Protein Score	Protein mass	Coverage	Unique peptide	Unique spectra	Description
IPI00220327	2405.85	66170.07	0.419	23	41	CK1
IPI00019359	2260.11	62254.9	0.531	25	45	CK9
IPI00021304	1715.65	66110.5	0.374	16	18	CK2
IPI00009865	1493.15	59019.78	0.341	16	25	CK10

Since the mass spectrometry analysis revealed that band 1 and 2 sequences were very similar, we hypothesized that the band 2 may be a degradation product of band 1. We thus used band 1 mainly as a reference for the subsequent experiments. On the basis of molecular size in the electrophoretogram, we initially surmised that CK10 and CK8 were more likely to be the target proteins 26. We therefore chose these two proteins for further analysis.

### Identification of candidate antigens by western blot

**Figure 4:**
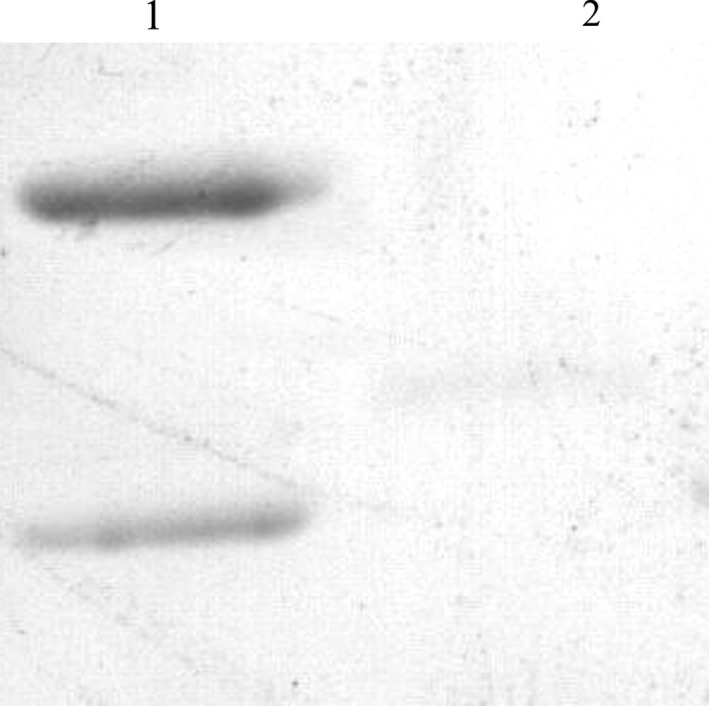
Western blotting analysis of the antigen:antibody reaction between the purified antigen and the commercial anti‐CK10 monoclonal antibody. Lane 1, CK10; Lane 2, CK8.

The specificity of the monoclonal antibody 11C3 for these antigens was detected by western blotting and compared to the specificities of the commercial anti‐CK10 and anti‐CK8 monoclonal antibodies. As shown in Fig. 4, the antigen:antibody reaction was observed between the total protein lysates from QGY‐7703 cells and the commercial anti‐CK monoclonal antibody (anti‐CK10 and CK8). Furthermore, a band at the corresponding position in the total protein lysates from QGY‐7703 cells was observed, and this band was not recognized by the commercial anti‐CK8 monoclonal antibody.

### Cytokeratin gene expression in hepatoma cells and normal liver cells

**Figure 5:**
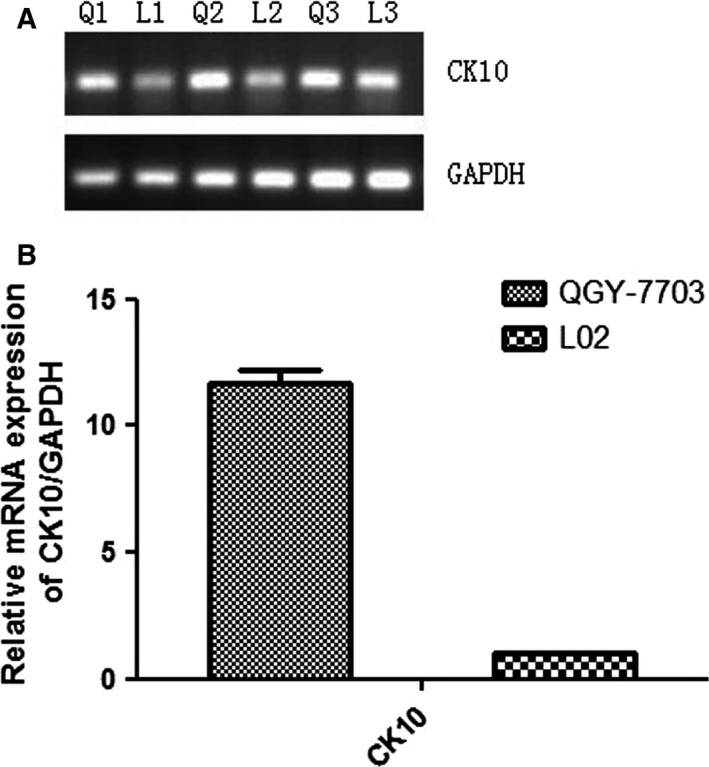
The analysis of relative expression level changes of CK10 mRNA in QGY‐7703 and L02. The GAPDH was used as the endogenous reference in three groups of parallel control experiments.

We examined CK10 mRNA expression by PCR, and found only products when CK10 was amplified. There were no products when CK8 was amplified. This is consistent with the results of western blot. Consistent with this, RT‐qPCR revealed that CK10 expression in hepatoma cells was 11.7‐fold higher than in normal liver cells (Fig. 5).

## Discussion

The premise and key of targeted cancer therapy is to obtain functional target molecules and target genes that exhibit tumor‐specific expression. This is particularly the case in HCC, where no markers that can be used for the detection of early‐stage diseases are available.

Cytokeratins constitute the largest family of the intermediate filament proteins, and are typical epithelial cell markers that are expressed in a tissue‐specific and differentiation‐dependent manner 27. Cytokeratins are widely expressed in a variety of malignant epithelial cells, and tend to be altered in association with increases in metastatic ability and malignancy 18, 19, 20. Differential expression of cytokeratins is often exploited for diagnostic purposes, such as for the identification of the primary organ site of malignancies and for differentiating malignant tissue from benign tissue 21, 22. To date, several cytokeratins have been used as prognostic indicators in many tumors of epithelial origin 23, 24, 25. For example, CK8/18 overexpression has been reported in hepatocellular carcinoma cells 26, while other studies have shown that high CK7 and CK9 expression correlates with tumor metastasis, and that these molecules could be used as biomarkers of the response to HCC treatment 28, 29, 30, 31.

Among the intermediate filament proteins that constitute the hepatocyte cytoskeleton, CK8 has been closely related to the occurrence and development of several hepatic diseases such as liver cirrhosis, virus hepatitis, and hepatocellular carcinoma 26, 32, 33, 34. CK10 is specific to stratified squamous epithelia and squamous cell carcinoma; however, relatively little is known about its role in cancer. Interestingly, however, CK10 overexpression has been correlated with tumor invasiveness, and could be a prognostic factor in epithelial tumors 35, 36.

In the study, we used Mascot to interrogate the IPI_human v.3.87 database to perform protein profiling. The results indicate that the antigen we identified is probably a cytokeratin, thereby implicating cytokeratins in the pathophysiology of hepatocellular carcinoma. Expression of CK10 was significantly higher in hepatoma carcinoma cells than in normal liver cells. Previous reports indicate that CK10 expression is associated with HCC invasiveness 35, 36. Our results are consistent with these reports, and we thus infer that CK10 may be involved in the formation and development of hepatocellular carcinoma, and may be a tumor‐associated antigen.

We note that the relationship between CK10 mRNA and protein expression is not strictly linear. Therefore, future studies will be designed to identify additional factor(s) that contribute to tumor‐specific upregulation of CK10 in HCC.

## Author contributions

ZZ and SZ wrote the paper. ZZ and ZL performed the experiments. YL and RL analyzed the data. CW and HL performed preliminary experiments. BG analyzed and interpreted the data. GL and SZ designed the experiments.
